# Supplementary material for: Development of a computer-aided design software for the quantitative evaluation of aesthetic damage
Source: PLoS One. 2019 Dec 18;14(12):e0226322. doi: 10.1371/journal.pone.0226322 (PMC6919621; doi:10.1371/journal.pone.0226322)
Supplement: S1 Code site — 1. (ZIP) [file pone.0226322.s001.zip › [EESC_jr][Projeto]Relatorio_07_21/reports/1/2017.07.28/21:49:22/report.pdf]

Preâmbulo

Na data de 29/07/2017 no horário 10:00, o(a) Perito(a) Sr. Rafael Yonezawa de Mello, Estudante, designado(a)para proceder ao exame pericial solicitado em: [nome] [sobrenome], do sexo Não informado, nascido(a) em 01/01/1990, ocupação [profissão], portador(a) do RG [documento] [órgão], cujo endereço [endereço], passa a expor a avaliação de dano estético corporal.

Exame objetivo da lesão

A lesão ora objeto de análise teve como natureza Acidente de trânsito, Acidente de trabalho, Violência interpessoal ocorrido em 01/01/2017. O prejuízo estético consiste em lesão cicatricial consolidada na região: Rosto, Costas, Superfície dos pés no lado superior.

Trata-se de cicatriz Quelóide de tamanho/frequência Única: Retilínea de orientação Horizontal de pigmentação Normocrômica medindo 10 centímetro(s) na horizontal, 1 centímetros na vertical e 10 milímetros de Profundidade. De contornos Regulares. A lesão tem repercussão é visível a 50 centímetros nas relações íntimas. A lesão é visível a 3 (três) metros nas relações sociais. A lesão tem repercussão na vida diária afetiva conforme fotos anteriores e atuais..

Conclui-se que classificada segundo os parâmetros de dano estético AIPE - Análise do Impacto do Prejuízo Estético (escala de seis níveis e pontuação entre 0 e 50 pontos), como sendo dano estético relativo à área 1, a lesão é caracterizada por 6 pontos e grau de prejuízo estético: Leve.

Escore final: 6;
Grau de prejuízo: Leve.

[local], 29/07/2017

____

Sr. Rafael Yonezawa de Mello
